# Serial magnetic resonance imaging and ultrasound examinations demonstrate differential inflammatory lesion patterns in soft tissue and bone upon patient-reported flares in rheumatoid arthritis

**DOI:** 10.1186/s13075-020-2105-6

**Published:** 2020-02-03

**Authors:** Dorota Kuettel, Daniel Glinatsi, Mikkel Østergaard, Lene Terslev, Jette Primdahl, Sören Möller, Andreas Pedersen, Randi Petersen, Ulrich Weber, Kim Hørslev-Petersen

**Affiliations:** 1 Danish Hospital for Rheumatic Diseases, University Hospital of Southern Denmark, Engelshøjgade 9A, DK-6400 Sønderborg, Denmark; 2 Department of Regional Health Research, University of Southern Denmark, Winsløwparken 19, C 5000 Odense, Denmark; 3 Department of Rheumatology, Skaraborg Hospital, Lövängsvägen, 541 42 Skövde, Sweden; 4 Copenhagen Center for Arthritis Research (COPECARE), Center for Rheumatology and Spine Diseases, Rigshospitalet, Glostrup, Denmark; 5 Department of Internal Medicine, University of Copenhagen, Nordre Ringvej 57, 2600 Glostrup, Denmark; 6 Hospital of Southern Denmark, University Hospital of Southern Denmark, Kresten Philipsens Vej 15, 6200 Aabenraa, Denmark; 7 OPEN - Open Patient data Explorative Network, Odense University Hospital and Department of Clinical Research, University of Southern Denmark, J.B. Winsløws Vej 9 A, C 5000 Odense, Denmark

**Keywords:** Patient-reported flares, RA, MRI, Ultrasonography

## Abstract

**Background:**

Magnetic resonance imaging (MRI) and ultrasonography (US) are more sensitive than clinical evaluation in assessing inflammation in rheumatoid arthritis (RA). Data is scarce regarding potential link between patient-reported flares and inflammation on imaging. The aim of the study was to explore the pattern and longitudinal associations of inflammatory lesions detected by serial MRI and US in relation to patient-reported flares in patients with RA.

**Methods:**

Eighty RA patients with baseline DAS28CRP < 3.2 and no swollen joints were examined at baseline and followed for 1 year. Patients were requested to contact the hospital in case of patient-reported hand flare accompanied by ≥ 1 tender and swollen joint. The 29 patients who reported hand flare had four extra visits within 4 months from flare onset comprising clinical examination, patient-reported outcomes, MRI, and US of wrists and hands. MRI synovitis/tenosynovitis/bone marrow edema (BME) and US synovitis/tenosynovitis were scored. MRI and US scores at and after the flare were compared to baseline before the flare, and associations were explored by linear mixed models for repeated measurements.

**Results:**

Synovitis and tenosynovitis by MRI/US increased significantly at flare onset. Synovitis waned quickly, as did US tenosynovitis. BME showed delayed increase yet persisted, once the patient-reported flare had resolved, as did MRI tenosynovitis. In univariate models, patient-reported flares were associated with all MRI and US inflammatory markers, except for BME, which was only associated with SJC28 and long-lasting flares > 14 days. Independent associations were observed between patient-reported flares and tenosynovitis by MRI and US (*p* < 0.05).

**Conclusions:**

Patient-reported flares were linked to inflammation detected by serial MRI and US. Differential patterns of inflammatory lesion evolution were observed by serial imaging with early synovial and tenosynovial inflammation, followed by delayed-onset BME.

## Background

Rheumatoid arthritis (RA) is characterized by substantial heterogeneity in both clinical presentation and disease course [1]. Flares, episodes of disease worsening, are frequently seen in RA [2]. Fluctuations in clinical disease activity may contribute to progression of radiographic joint damage and impact co-morbidity [3, 4]. Given the claim to incorporate the patients’ perspective during development of a flare definition, the concept of patient-reported flare has emerged [5, 6]. Patient-reported flares have an impact on everyday life [7], but there is scarce evidence indicating as to whether they are linked to objectively assessed immune-mediated inflammation, functional impairment, and radiographic progression [8].

Imaging modalities such as magnetic resonance imaging (MRI) and ultrasonography (US) are superior to clinical assessment for detecting inflammation and are recommended by the European League Against Rheumatism (EULAR) for more accurate evaluation of inflammation [9]. Several hypotheses about origin and evolution of immune-mediated inflammation in RA pathogenesis have been proposed [10].The sequence of tissue inflammation during a flare in RA is unknown. Subclinical inflammation may be present at the state of clinical remission [11–13]. Clinical flares may be precipitated by incomplete suppression of inflammation, as US-detected persistent synovitis has been shown to predict clinical flares [13–15].

There is limited evidence about the relation between patient-reported flare and inflammatory lesions on imaging. A cross-sectional study reported US tenosynovitis to be associated with the FLARE-RA questionnaire, which is designed to detect flares between clinical visits [16]. There are no published data investigating a potential link between patient-reported flares and inflammation detected by MRI.

The aim of our study was to explore the pattern and longitudinal associations of inflammatory lesions detected by serial MRI and US in relation to patient-reported flares in patients with RA in low disease activity (LDA) state.

## Methods

### Study design and participants

Eighty consecutive patients, recruited from August 2016 to June 2017, from the outpatient clinic at the Danish Hospital for Rheumatic Diseases, Sønderborg, Denmark, established the overall FLAre-in-RA (FLARA) cohort. Patients aged ≥ 18 years were eligible if they met the American College of Rheumatology (ACR) 1987 or ACR/EULAR 2010 criteria for RA, were anti-cyclic citrullinated peptide antibody (anti-CCP) and/or rheumatoid-factor (RF) positive, had a Disease Activity Score based on C-reactive protein (DAS28CRP) at baseline < 3.2 and no swollen joints, were on stable disease modifying anti-rheumatic drugs (DMARD) treatment with no intra-articular glucocorticoid injections 4 weeks prior to study entry, and had no contraindications for MRI.

**Fig. 1:**
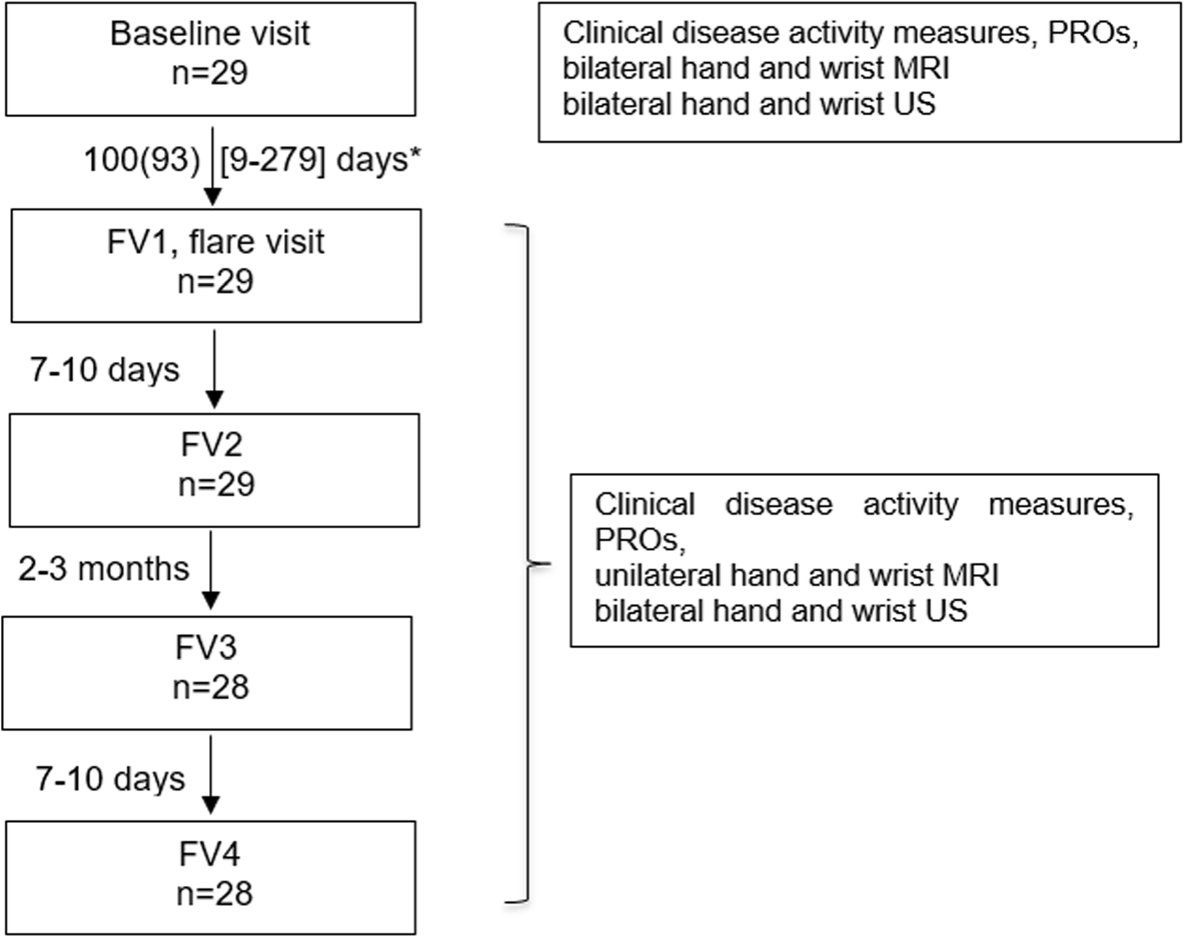
Flowchart of the patients’ path and overview of procedures/data collected and intervals between visits. FV, follow-up visit; MRI, magnetic resonance imaging; PRO, patient-reported outcome; US, ultrasonography. *Mean (SD) time interval between baseline and the flare visit, which was initiated by a patient, who contacted hospital because of a patient-reported hand flare

The cohort was followed for 1 year. Patients were requested to contact the outpatient clinic in case of a flare in a hand or wrist (a “hand flare”) accompanied by at least one tender and swollen joint, as perceived by the patient. All 80 patients underwent clinical, laboratory, MRI, and US examinations at baseline. Patients with hand flares were included in the present study and underwent assessments at four extra follow-up visits (FV1-FV4) (Fig. 1). The first immediate follow-up visit (FV1), also called the flare visit, was scheduled within 72 h upon patient’s contact to the hospital. Seven to 10 days later, patients were seen at the second follow-up visit (FV2). Finally, after 2–3 months, the patients were assessed twice with 7 to 10 days interval at the follow-up visits 3 and 4 (FV3 and FV4).

Intra-articular or intra-muscular glucocorticoid injections were only allowed at FV2 and FV4 once clinical evaluation and imaging procedures were completed.

### Clinical, biochemical, and patient-reported outcomes

At baseline, information about age, gender, disease duration, and ongoing therapy for RA was collected. The patients were tested for RF and anti-CCP positivity. At each visit, a senior rheumatologist or a trained study nurse, who were blinded to imaging findings and patient-reports, carried out clinical assessment for 28 swollen (SJC28) and tender joints (TJC28). High-sensitive CRP (mg/l) level was determined to calculate DAS28CRP. Patients indicated swollen and tender joints on a mannequin format. Visual analog scales (VAS) were utilized for evaluation of pain, patient’s and evaluator’s global assessments (PGA and EGA, respectively). The Danish version of the Health Assessment Questionnaire (HAQ) was applied to assess physical function. Patient-reported flare was defined by the anchor question “Are you experiencing a flare of your RA at this time?” (yes/no) [17, 18]. Patients filled in the OMERACT (Outcome Measures in Rheumatology) Rheumatoid Arthritis Flare Questionnaire (RA-FQ) [17, 18]. Patients reporting flare rated flare severity on a scale from 0 to 10 and reported flare duration according to four categories: 1–3, 4–7, 8–14 or > 14 days. As the reports were collected at each follow-up visit, the flare duration category may have changed in an individual patient as a consequence of the increasing observation period. Moreover, information about date for hand flare onset and termination was collected.

### MRI

An ONI OrthOne 1.0 Tesla (T) MR unit was used for all MRI examinations. At baseline, MRIs of the wrists and bilateral second to fifth metacarpophalangeal (MCP) and proximal interphalangeal (PIP) joints were conducted. At each FV, a unilateral MRI of wrist and hand was repeated on the side of the initial patient-reported flare. In case of flare in both hands, the most affected side, according to the patient, was chosen. Regarding baseline MRIs, only unilateral scans of the side examined at follow-up visits were included in the analyses. A coronal T1-weighted three-dimensional gradient echo sequence (T1w 3D GE), allowing multiplanar reconstruction, before and after gadolinium contrast injection [0.1 mmol gadoteric acid per kg body weight] and a coronal short-tau inversion recovery (STIR) sequence before contrast injection were acquired, following the recommendations of the OMERACT RA MRI Scoring system (RAMRIS) [19].

Parameters of the MRI sequences were as follows: for T1w 3D GE: flip angle 25°, repetition time (TR) 40 milliseconds (ms); echo time (TE) 18 ms, slice thickness (ST) 0.8 mm (mm); matrix 216 × 216, and field of view (FOV) 100 mm; for STIR: flip angle 90°, TR 4100 ms; TE 40 ms, ST 3 mm; matrix 256 × 256, and FOV 160 mm.

Two readers, one experienced (DG) and one newly trained (DK) in RAMRIS scoring, blinded to the chronology, clinical, laboratory, patient-reported outcomes (PROs), and US imaging data, evaluated the MRIs. Scans from the five time points were read simultaneously for inflammatory lesions, i.e., synovitis, bone marrow edema (BME), and tenosynovitis according to the RAMRIS [19, 20]. Synovitis was assessed in three wrist regions (the distal radioulnar joint, the radiocarpal joint, and the intercarpal and carpometacarpal joints) and in the second to fifth MCP and PIP joints, respectively, on a scale 0–3. Tenosynovitis in the wrist was assessed separately at extensor tendon compartment I-VI and three flexor tendon compartments, and in the hands, the second to fifth flexor tendons were assessed at the level of the MCP and PIP joints, respectively [19, 20]. Each bone was scored separately for BME on a scale 0–3 based on the proportion of bone volume affected by BME [19]. Sum scores for MRI synovitis, tenosynovitis, and BME, respectively, were calculated. The average scores from the two readers were used in the analyses.

For intra-reader agreement analysis, scans of five patients from all time points were re-anonymized and rescored.

### US examination

A General Electric Logiq E9 US machine with a multifrequency linear array transducer 6-15 was used for all examinations with unchanged Color Doppler (CD) settings throughout the study with Doppler frequency 7.5 MHz, pulse repetition frequency of 0.4 MHz, and Doppler gain just below the noise threshold, for detection of slow flow according to recommendations [21]. US examinations were conducted at baseline and each FV. The US examiner was blinded to the clinical, laboratory, MRI, and patient-reported data. The US protocol included multiplanar scanning of 22 joints/regions: bilateral wrists (radiocarpal, midcarpal, and distal radioulnar joints, dorsal recesses), the first to fifth MCP joints, dorsal recesses, the first interphalangeal (IP) joint, the second to fifth PIP joints, dorsal and volar recesses, extensor tendon compartments I–VI, three flexor tendons/groups (flexor carpi radialis, flexor pollicis longus, and combined flexor digitorum superficialis and profundus), and finger flexors of the second through fifth finger. Finger flexors were evaluated in a manner to assess the whole synovial sheath-covered area of the tendons and were scored only once, without distinction between the level of MCP and PIP. Synovitis and tenosynovitis were defined according to the OMERACT definitions, assessed by CD and gray scale (GS) and graded semi-quantitatively 0–3 [22–25]. One combined GS and Doppler OMERACT-EULAR score was generated for synovitis and tenosynovitis [23, 24]. The scores from single joints/regions were added into a Global OMERACT-EULAR Synovitis Score (GLOESS) [26]. For the wrist, one single score was used, corresponding to the maximum combined score from any of the joints evaluated at this region. Per analogy, scores from single tendons/tendon compartments were added into a total tenosynovitis score.

### Variables for the analyses

Inflammatory lesions by imaging sum scores of synovitis, tenosynovitis, and BME, respectively, on MRI and GLOESS and sum score of tenosynovitis on US, constituted dependent variables. Patient-reported flare was the main explanatory variable. The choice of covariates was based on external evidence on associations with imaging inflammation found in previous studies, i.e., CRP and SJC28 [16, 27], and among PROs pain, PGA and HAQ [28, 29]. Due to the limited sample size, no additional covariates were included.

### Statistical analysis

Data is reported as mean (SD) or numbers (%), as appropriate. All outcomes assessed at the follow-up visits were compared to baseline using *t* tests or Wilcoxon signed-rank tests, depending on the distribution of the data as evaluated by quantile plots. The evolution of inflammatory changes by the two imaging modalities was illustrated by mean plots.

We compared all outcomes at FV3 and FV4 in non-flaring patients, to explore whether the outcomes differed when measured 1 week apart.

Because of the data structure with serial measurements on the same individuals, linear mixed models were utilized, as they explicitly allow for clustering of observations from the same individual and analyzed associations across all time points simultaneously. We included a random effect for each patient in all the models. Our analysis plan was based on three different scenarios: a univariate, a full multivariate model, and a final model after backward selection from the full model.

Primarily, series of univariate models were fitted for each explanatory variable using MRI or US inflammatory markers as the dependent variable. As a second step, all explanatory variables were included in the full multivariate model with age, sex, and disease duration at baseline as possible confounders. Finally, we conducted a backward selection from the full model with a 0.05 *p* value cutoff to reach the final models. As a sensitivity analysis, flare duration (four categories) was used as an explanatory variable.

The validity of the models’ assumptions was tested, checking variance homogeneity and normality of random effects and residuals by diagnostic plots. Logarithmic transformations of outcomes were applied if appropriate and collinearity was checked for in the multivariate models. As mixed effects regression models take into account missing outcome observations by design, we did not otherwise model missing data.

For MRI reliability assessment, both inter-reader and intra-reader agreement analyses utilized intraclass correlation coefficients (ICCs; two-way mixed effects model, absolute agreement). Moreover, the smallest detectable change (SDC) was calculated for the change in score between baseline and FV1.

Coefficients are reported with 95% confidence interval (95% CI). *p* values < 0.05 were considered statistically significant. The analyses were carried out using Stata 15.0 (StataCorp, TX, USA).

## Results

A total of 80 RA patients were included in the FLARA cohort. Twenty-nine of 80 patients (36%) contacted the hospital because of a hand flare and formed the sample for the present study. Patient flow and overview of procedures at each visit and intervals between visits are presented in Fig. 1.

In total, 143 MRI and US examinations were performed. Since two patients declined contrast administration during MRI at FV3 and FV4, the number of MRI assessments of synovitis and tenosynovitis was 139. Except one missing HAQ at FV4, there were no missing values for clinical or PRO variables included in the main analyses.

### Patients’ characteristics, clinical findings, and PROs

Patients’ demographic and clinical characteristics at baseline and follow-up visits are reported in Table 1. At baseline, 22/29 patients (76%) were in DAS28CRP remission (DAS28CRP < 2.6) and 27/29 (93%) of the patients received conventional synthetic DMARD (csDMARD), two of them with concomitant biologic DMARD (bDMARD), 2/29 (7%) patients did not receive any DMARD, and none received glucocorticoid therapy. None of the patients changed their arthritis medication between baseline and the flare visit. During follow-up, 2/3/4/3 patients, escalated or changed csDMARD at FV1/FV2/FV3/FV4, respectively. None of the patients initiated or changed bDMARD, and one patient started oral glucocorticoid at FV3. Four and three patients were treated with intra-articular glucocorticoid injections at FV2 and FV4, respectively, after imaging procedures were carried out.

**Table 1:** Characteristics at baseline and follow-up visits of 29 RA patients reporting hand flare during 1 year of follow-up

	Baseline (*n* = 29)	FV1 (flare visit) (*n* = 29)	FV2 (*n* = 29)	FV3 (*n* = 28)	FV4 (*n* = 28)
No. reporting flare	0/29	29/29	22/29	3/27	6/27
Age, years	64.8 (9.7)	NA	NA	NA	NA
Female/male (%)	20/9 (69/31)	NA	NA	NA	NA
Disease duration, years	10.3 (5.5) [2–21]	NA	NA	NA	NA
Anti-CCP positive (%)	27 (93)	NA	NA	NA	NA
IgM RF positive (%)	26 (90)	NA	NA	NA	NA
DAS28CRP	2.0 (0.7) [1.2–3.1]	3.5 (1.0)*** [1.2–5.3]	3.0 (1.2)*** [1.2–6.0]	2.4 (0.9) [1.2–4.9]	2.6 (1.0)* [1.2–4.9]
CRP (mg/l)	5.7 (8.6) [0–33]	10.1 (12.7)*** [0.4–48]	6.8 (9.2) [0–44]	6.1 (6.9) [0.4–23]	6.8 (11.4) [0.4–56]
SJC28	0 (0) [0–0]	1.5 (1.0)*** [0–4]	1.0 (1.4)*** [0–5]	0.2 (0.5) [0–2]	0.4 (0.7)** [0–3]
TJC28	0.8 (1.5) [0–7]	4.0 (3.5)*** [0–12]	3.8 (4.9)*** [0–21]	1.6 (2.0) [0–8]	1.9 (2.1)** [0–7]
EGA (VAS)	4.1 (3.2) [0–11]	16.2 (13.1)*** [2–74]	11 (9.9)*** [0–47]	5.8 (5.8) [0–27]	6.5 (5.8) [0–26]
PROs					
Pain (VAS)	16.7 (18.4) [0–71]	43.2 (22.8)*** [0–74]	36.4 (26.7)** [0–98]	24.2 (23.7) [0–96]	25.8 (22.9) [0–74]
PGA (VAS)	22.1 (23.1) [0–84]	43.4 (26.2)*** [0–85]	37.1 (26.2)** [0–98]	26.3 (24.3) [0–93]	26.6 (26.0) [0–80]
Patient-reported TJC28	1 (1.9) [0–8]	4.0 (3.0)*** [1–11]	2.5 (3.5)* [0–14]	1.4 (2.6) [0–13]	1.8 (2.8) [0–12]
Patient-reported SJC28	0 (0) [0–0]	2.8 (2.9)*** [1–11]	1.9 (3.1)*** [0–12]	0.7 (1.8)** [0–8]	1 (2.1)** [0–10]
HAQ	0.4 (0.4) [0–1.5]	0.6 (0.5)*** [0–1.5]	0.5 (0.5) [0–1.8]	0.4 (0.5) [0–1.9]	0.5 (0.5) [0–1.9]
Patient-reported flare characteristics					
RA-FQ (0–50)	10.1 (9.6) [0–33]	22.9 (12.7)*** [1–49]	15 (10.7) [1–37]	9.5 (8.6) [0–35]	10.5 (9.9) [0–38]
Flare severity^a^ (0–10)	NA	4.8 (2.7) [1–10]	2.67 (2.4)*** [0–8]	1.25 (2.3)** [0–9]	1.45 (2.3)** [0–7]
Flare duration (*n*)					
1–3 days	NA	18	4	1	4
4–7 days	NA	8	10	0	0
8–14 days	NA	2	0	0	0
> 14 days	NA	1	4	2	2
MRI					
Synovitis	10.3 (4.0) [0–17.5]	12.7 (3.1)*** [7–19]	11.9 (3.2)** [6–19]	11.0 (3.7) [3–18.5]	11.5 (3.6) [4.5–20]
Tenosynovitis	6.4 (4.7) [0–17.5]	9.3 (5.8)*** [1–20.5]	8.1 (4.9)** [1–19.5]	7.9 (5.2)** [0–20.5]	8.2 (4.8)** [0–20.5]
BME	2.6 (4.8) [0–22.5]	2.6 (3.1) [0–11]	2.9 (3.6)** [0–11.5]	2.9 (3.5)** [0–12]	2.9 (3.5)* [0–11.5]
No. (%) with synovitis sum score ≥ 1	28 (97)	29 (100)	29 (100)	26 (100)	26 (100)
No. (%) with tenosynovitis sum score ≥ 1	28 (97)	29 (100)	29 (100)	25 (96)	26 (100)
No. (%) with BME ≥ 1	14 (48)	20 (69)	18 (62)	18 (64)	17 (61)
US					
Synovitis (GLOESS)	8.3 (5.4) [0–23]	11.3 (5.3)* [3–25]	10.6 (5.0)* [3–23]	9.7 (5.0) [3–23]	10.04 (5.1) [3–23]
Tenosynovitis	3.5 (4.6) [0–14]	6.3 (6.2)*** [0–19]	5.9 (5.9)** [0–24]	4.4 (5.0) [0–15]	4.6 (5.0) [0–15]
No.(%) with GLOESS ≥ 1	28 (97)	29 (100)	29 (100)	28 (100)	28 (100)
No.(%) with tenosynovitis sum score ≥ 1	18 (62)	20 (69)	23 (79)	19 (68)	19 (68)

Mean (SD) [range] or numbers (%) of demographic, clinical, patient-reported, and imaging characteristics. *Anti-CCP* anti-cyclic citrullinated peptide antibody, *BME* bone marrow edema, *CRP* C-reactive protein, *DAS* disease activity score, *EGA* evaluator global assessment, *FV* follow-up visit, *GLOESS* Global OMERACT (Outcome Measures in Rheumatology) –EULAR European League Against Rheumatism) Synovitis Score, *HAQ* health assessment questionnaire, *MRI* magnetic resonance imaging, *PGA* patient’s global assessment, *PROs* patient-reported outcomes, *RA-FQ* OMERACT Rheumatoid Arthritis Flare Questionnaire, *RF* Rheumatoid factor, *SJC28* swollen joint count in 28 joints, *TJC28* tender joint count in 28 joints, *US* ultrasonography, *VAS* visual analog scale; *p* value for paired data comparison between baseline and each follow-up visit assessed by paired *t* test or Wilcoxon signed-rank test, as appropriate

***p ≤ 0.001, **p ≤ 0.01, *p < 0.05

^a^For variable flare severity comparisons are made vs FV1, as no patient reported flare at baseline

After contacting the outpatient clinic because of a hand flare, 16/29 (55%) patients were seen at FV1 the same day, while 10/2/1 patients were scheduled within 1/2/3 days from the telephone contact, respectively. The mean (SD) duration of the hand flare was 12 (13.9) days, ranging between 3 and 66 days. More than half of the patients, 16/29 (55%), reported bilateral swollen joints at the flare visit, and 18/29 (62%) reported bilateral tender joints. At the flare visit, 25/29 (86%) patients had ≥ 1 swollen joint as evaluated by the physician.

### Evolution of inflammatory lesions by MRI and US before, at, and after patient-reported flare

At flare visit (FV1), imaging markers of inflammation, except for BME, had increased significantly compared to baseline, concordant with clinical measures of disease activity and PROs (Table 1).

**Fig. 2:**
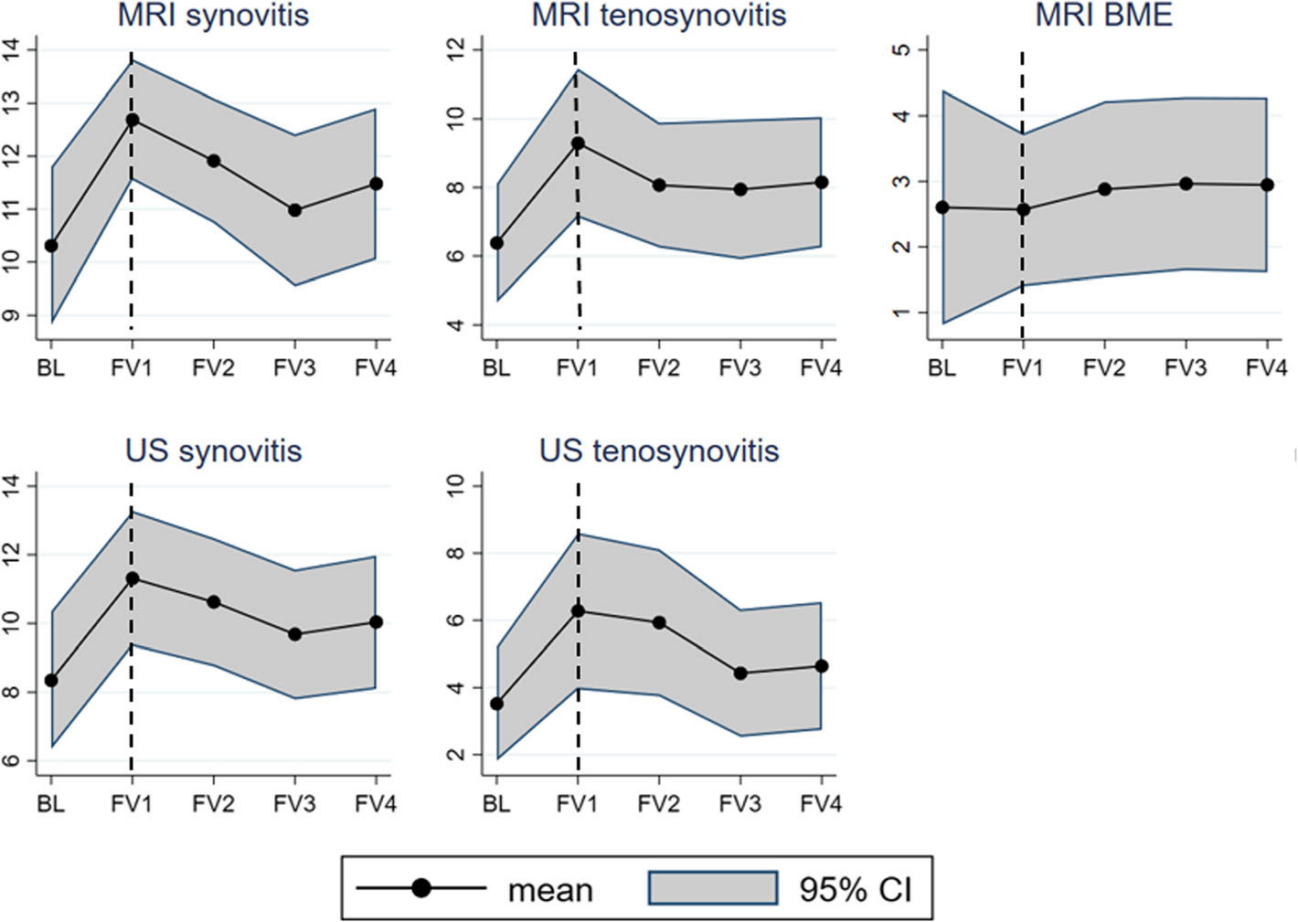
Evolution of imaging-assessed inflammation over time. BL, baseline, BME, bone marrow edema; 95%CI, 95% confidence interval; FV, follow-up visit; MRI, magnetic resonance imaging; US, ultrasonography. FV1 is the visit at the time of patient-reported hand flare. FV2 took place 7–10 days after FV1. FV3 took place 2–3 months after FV2. FV4 took place 7–10 days after FV3. The vertical dashed line marks the time of patient-reported flare

At FV2, imaging parameters, except for BME, decreased, with fewer patients reporting flare (*n* = 22), paralleled by decreasing clinical disease activity measures and PROs. By contrast, BME increased significantly as compared to baseline and remained elevated at FV3 and FV4 after the hand flare had resolved (*p* < 0.05). Similarly, MRI tenosynovitis stayed significantly increased at FV3 and FV4, as compared to baseline (*p* < 0.01) (Table 1). The sequential changes in specific inflammatory lesions on MRI and US are presented graphically in Fig. 2.

After the initial hand flare had resolved, three patients reported a new flare at FV3. In order to elucidate whether the subsequent flares may drive the persistent elevation in BME and MRI tenosynovitis score, we conducted sensitivity analyses by comparing imaging scores from baseline to FV3 after exclusion of the three patients with a second flare. The results did not diverge from the main analyses: MRI tenosynovitis and BME, respectively, were significantly elevated at FV3 as compared to baseline (*p* < 0.05) (Additional file 1: Table S1).

### Changes in clinical, PROs, and imaging biomarkers when not reporting flare

We did not observe significant differences in any of the outcomes, except for PGA (*p* = 0.03), when comparing assessments obtained 7–10 days apart, at FV3 and FV4, in patients without a flare at these visits (*n* = 21) (Table 2).

**Table 2:** Characteristics of 21 patients who did not report a flare at the third and fourth follow-up visit

Characteristic	Follow-up visit 3	Follow-up visit 4	*p* value
DAS28CRP	2.2 (0.7)	2.2 (0.7)	0.59
CRP (mg/l)	5.2 (6.1)	5.1 (7,0)	0.82
SJC28	0.1 (0.4)	0.1 (0.4)	0.36
TJC28	1.5 (2.2)	1.5 (2.1)	0.64
EGA (VAS)	4.7 (4.3)	4.8 (3.6)	0.74
Pain (VAS)	16.2 (15.3)	15.7 (13.1)	0.76
PGA (VAS)	18.9 (17.5)	15.2 (16.3)	0.03
Patient-reported TJC28	1.0 (1.5)	1.0 (1.9)	0.80
Patient-reported SJC28	0.4 (1.1)	0.3 (0.7)	0.98
HAQ	0.4 (0.5)	0.4 (0.5)	0.22
RA-FQ	6.9 (5.3)	7.0 (6.6)	0.95
MRI synovitis	11.0 (3.5)	11.5 (3.6)	0.92
MRI tenosynovitis	6.6 (4.6)	6.8 (4.4)	0.46
MRI BME	3.3 (3.7)	3.3 (3.7)	0.19
GLOESS	9.1 (4.3)	9.4 (4.5)	0.08
US tenosynovitis	3.6 (4.5)	4.1 (5.1)	0.23

Mean (SD) of clinical, patient-reported, and imaging characteristics. *BME* bone marrow edema, *CRP* C- reactive protein, *DAS* disease activity score, *EGA* evaluator global assessment, *GLOESS* Global OMERACT (Outcome Measures in Rheumatology) -EULAR (European League Against Rheumatism) Synovitis Score, *HAQ* health assessment questionnaire, *MRI* magnetic resonance imaging, *PGA* patient’s global assessment, *RA-FQ* OMERACT Rheumatoid Arthritis Flare Questionnaire, *SJC28* swollen joint count in 28 joints, *TJC28* tender joint count in 28 joints, *US* ultrasonography, *VAS* visual analog scale; *p* value for paired data comparison between visit 3 and visit 4 assessed by paired *t* test or Wilcoxon signed-rank test, as appropriate

### Reliability of scoring MRI and US

Inter- and intra-reader ICCs and SDCs for MRI sum scores of synovitis, tenosynovitis, and BME are presented in Additional file 2: Table S2 and Additional file 3: Table S3, respectively. All ICCs were > 0.80. Reliability analyses of US scoring have been published previously, showing very high agreement for all assessed pathologies [30].

### Associations with inflammation detected by MRI across all time points

The univariate analyses showed associations for MRI synovitis and tenosynovitis with CRP (*p* < 0.01), SJC28 (*p* ≤ 0.01), patient-reported flare (*p* < 0.0001), pain (*p* ≤ 0.001), and PGA (*p* < 0.05) (Table 3). BME scores were only significantly associated with SJC28 (*p* = 0.017).

**Table 3:** Associations between clinical and patient-reported outcomes and MRI-detected inflammation: synovitis, tenosynovitis, and BME

	Univariate model		Full multivariate model		Multivariate model after backward selection	
	Coefficient (95% CI)	*p*	Coefficient (95% CI)	*p*	Coefficient (95% CI)	*p*
MRI synovitis						
Flare	1.60 (0.88; 2.31)	< 0.0001	0.82 (− 0.17; 1.80)	0.10		
SJC28	0.96 (0.61; 1.32)	< 0.0001	0.74 (0.29; 1.17)	0.001	0.97 (0.62–1.32)	< 0.0001
CRP	0.09 (0.03; 0.16)	0.004	0.05 (− 0.02; 0.12)	0.18		
Pain (VAS)	0.03 (0.01; 0.05)	0.001	0.03 (− 0.00; 0.07)	0.14		
PGA (VAS)	0.02 (0.00; 0.04)	0.03	− 0.04 (− 0.07; 0.00)	0.08		
HAQ	0.70 (− 0.57; 1.98)	0.28	− 0.32 (− 1.80; 1.15)	0.67		
MRI tenosynovitis						
Flare	1.79 (0.99; 2.59)	< 0.0001	1.15 (0.05; 2.25)	0.04	1.15 (0.12–2.18)	0.029
SJC28	0.70 (0.27; 1.12)	0.012	0.02 (− 0.50; 0.50)	0.95		
CRP	0.16 (0.08; 0.24)	< 0.0001	0.15 (0.06; 0.23)	< 0.0001	0.15 (0.07–0.22)	< 0.0001
Pain (VAS)	0.04 (0.02; 0.06)	0.0001	0.06 (0.02; 0.10)	0.006	0.06 (0.02–0.10)	0.004
PGA (VAS)	0.03 (0.01; 0.05)	0.013	− 0.06 (− 0.10; − 0.01)	0.009	− 0.06 (− 0.10–0.02)	0.006
HAQ	1.00 (− 0.46; 2.52)	0.18	0.15 (−1.60; 1.90)	0.87		
MRI BME*						
Flare	0.05 (− 0.05; 0.15)	0.29	− 0.70 (− 0.21; 0.07)	0.32		
SJC28	0.06 (0.01; 0.11)	0.017	0.07 (0.01; 0.14)	0.02	0.06 (0.01–0.11)	0.017
CRP	0.01 (0.00; 0.01)	0.21	− 0.00 (− 0.01; 0.00)	0.63		
Pain (VAS)	0.00 (0.00; 0.00)	0.16	0.00 (− 0.00; 0.01)	0.53		
PGA (VAS)	0.00 (0.00; 0.00)	0.17	0.00 (− 0.01; 0.01)	0.94		
HAQ	0.09 (− 0.09; 0.27)	0.33	− 0.03 (− 0.28; 0.91)	0.75		

*BME* bone marrow edema, *CRP* C-reactive protein, *HAQ* health assessment questionnaire, *MRI* magnetic resonance imaging, *PGA* patient’s global assessment, *SJC28* swollen joint count assessed in 28 joints, *VAS* visual analog scale. All multivariate mixed effect linear regression models are adjusted for age, sex, and disease duration at baseline. *log transformed

In the multivariate models, SJC28 but no PROs remained significantly associated with MRI synovitis (*p* ≤ 0.001) or BME (*p* ≤ 0.02) (Table 3). MRI tenosynovitis was independently associated with patient-reported flare, also after backwards selection (*p* < 0.05). CRP (*p* < 0.0001), pain (*p* = 0.004), and PGA (*p* = 0.006) also demonstrated independent associations with MRI tenosynovitis (Table 3).

### Associations with inflammation detected by US across all time points

The univariate analyses showed significant associations between GLOESS and patient-reported flare (*p* = 0.01), SJC28 (*p* = 0.01), and CRP (borderline significant *p* = 0.056), while independent associations were only observed for SJC28 (*p* = 0.009) (Table 4). Patient-reported flare (*p* = 0.0015) and SJC28 (*p* = 0.0003) were associated with US tenosynovitis in the univariate and full multivariate models (*p* < 0.05). HAQ, pain, and PGA did not demonstrate any associations with US parameters (Table 4).

**Table 4:** Associations between clinical and patient-reported outcomes and US-detected inflammation: synovitis (GLOESS) and tenosynovitis

	Univariate model		Full multivariate model		Multivariate model after backward selection	
	Coefficient (95% CI)	*p*	Coefficient (95% CI)	*p*	Coefficient (95% CI)	*p*
US synovitis (GLOESS)						
Flare	1.58 (0.35; 2.82)	0.01	1.12 (− 0.58; 2.80)	0.19		
SJC28	0.81 (0.19; 1.43)	0.01	0.78 (0.00; 1.50)	0.049	0.83 (0.20;1.45)	0.009
CRP	0.09 (− 0.01; 0.18)	0.056	0.02 (− 0.09; 0.12)	0.73		
Pain (VAS)	0.02 (− 0.02; 0.05)	0.33	0.006 (− 0.05; 0.07)	0.85		
PGA (VAS)	0.01 (− 0.02; 0.04)	0.55	− 0.02 (− 0.09; 0.04)	0.45		
HAQ	− 0.23 (− 2.24; 1.78)	0.82	− 0.83 (− 3.27; 1.60)	0.50		
US tenosynovitis						
Flare	1.44 (0.55; 2.32)	0.0015	1.35 (0.08; 2.61)	0.037		
SJC28	0.82 (0.37; 1.27)	0.0003	0.70 (0.14; 1.27)	0.015	0.82 (0.38; 1.27)	< 0.0001
CRP	0.09 (0.01; 0.16)	0.03	0.03 (− 0.05; 0.12)	0.41		
Pain (VAS)	0.01 (− 0.01; 0.04)	0.23	− 0.01 (− 0.06; 0.03)	0.57		
PGA (VAS)	0.01 (− 0.01; 0.04)	0.32	− 0.01 (− 0.05; 0.04)	0.80		
HAQ	− 0.08 (− 1.70; 1.54)	0.92	− 1.02 (− 3.00; 0.94)	0.30		

*CRP* C-reactive protein, *GLOESS* Global OMERACT (Outcome Measures in Rheumatology)-EULAR (European League Against Rheumatism) Synovitis Score, *HAQ* health assessment questionnaire, *PGA* patient’s global assessment, *SJC28* swollen joint count assessed in 28 joints, *US* ultrasonography, *VAS* visual analog scale; all multivariate mixed effect linear regression models are adjusted for age, sex, and disease duration at baseline

### Associations between imaging biomarkers and flare duration across all time points

In the sensitivity analyses after adjusting for sex, age, and disease duration, MRI synovitis (*p* = 0.003), BME (*p* = 0.016), and US synovitis (*p* = 0.04) were significantly higher in the long-lasting flares (> 14 days) than in the short-lived flares (Additional file 4: Table S4).

## Discussion

We conducted the present study with serial MRI and US scans prior to, at the time of, and after patient-reported flare to explore the longitudinal relationship between inflammation detected by sensitive imaging modalities and patient-reported flares. Patient-reported flares were significantly associated with MRI- and US-detected synovitis and tenosynovitis, the latter showing independent associations. Long-lasting flares > 14 days were independently associated with synovitis by MRI and US as well as with BME. Sequential analysis of inflammatory imaging changes displayed a differential lesion pattern in relation to flare dynamics: MRI and US synovitis increased early at flare onset and resolved quickly, alike US tenosynovitis. BME evolved with delay, and remained for months, while MRI tenosynovitis raised promptly and persisted after the flare waned.

Among the assessed PROs, patient-reported flare showed an association with tenosynovitis detected by MRI or US. The association was independent of other PROs and independent of clinical disease activity expressed by CRP and SJC28. By contrast, synovitis detected by MRI or US was not independently associated with flare. Historically, synovitis has gained more attention than tenosynovitis, even though inflammation of the synovium, a hallmark of RA, affects not only joints but also tendon sheaths [16, 31–33]. A study of a large cohort of patients with established RA in remission found cross-sectional associations between US Power Doppler tenosynovitis and the flare self-assessment tool, FLARE-RA [16]. These findings are virtually concordant with our observations, even though we utilized a different definition of flare. In agreement with our study, no associations were found between US synovitis and the FLARE-RA [16]. Another study in RA patients with active disease and US-verified tenosynovitis reported that patient’s VAS for tenosynovitis pain was responsive to treatment, parallel to improvement in US parameters, indicating that patients’ pain perception may be triggered by tenosynovitis alone [34]. There is growing evidence that imaging-detected tenosynovitis has impact on RA diagnosing and prognostication of radiographic outcomes and prediction of clinical flares [35–38]. Our findings add to this evidence and lend support to the importance of tenosynovitis assessment at flares. While clinical examination focuses on joint counts, pain induced by tenosynovitis might be more relevant from the patients’ perspective.

To the best of our knowledge, this is the first study to explore associations between patient-reported flares and inflammatory markers on MRI in patients with RA. Previous studies demonstrated associations between MRI inflammation and PROs such as pain, PGA, and HAQ in early and active RA [28, 29, 39]. One previous study assessed the relationship between MRI-detected joint inflammation and pain, PGA, and physical function in patients with established RA in remission and at relapse [40]. Contrary to our results, inflammatory lesions were not associated with any of the PROs, neither in remission nor in relapse. Relapse was defined by DAS28CRP criterion, which precludes direct comparison with our data. In our study, the associations were weak between MRI synovitis and pain and PGA, respectively, and no associations were observed after adjusting for covariates. For MRI tenosynovitis, we found independent associations with pain and PGA. However, the magnitude of coefficients was low, signifying weak associations of limited clinical significance or even inverse association in case of PGA showing lower levels of MRI tenosynovitis with increasing PGA.

We did not find any associations between US and pain or PGA. However, effect estimates for MRI and US associations cannot be compared directly. The regions assessed did not entirely overlap as we carried out a bilateral hand/wrist US examination while we only performed unilateral MRI of the side reported by the patient as affected by flare. This might have strengthened the associations between MRI and PROs. However, even though patients had to decide which side was most affected by a flare in order to perform unilateral MRI due to feasibility and logistic reasons, the majority of the patients reported bilateral symptoms: 55% reported bilateral swollen joints at the flare visit, and even a higher proportion (62%) reported bilateral tender joints.

Nevertheless, we found a concordant pattern of associations with SJC28, as swollen joints were independently associated with synovitis both on MRI and US, suggesting that swollen joints reflect synovitis.

Whether RA starts as a primary inflammation of the synovium and subsequently spreads into the bone marrow, or vice versa, remains unanswered [10]. Similarly, the anatomic structure initially affected by inflammation at flare onset is unknown. Our results point towards the “outside-in” hypothesis, where the inflammatory process initially flares up in the synovium, as both synovitis and tenosynovitis scores increased immediately upon patient-reported flare, while BME evolved with delay. Interestingly, BME and MRI tenosynovitis persisted after the flare waned. BME is known as a strong predictor of radiographic progression in RA patients in remission [41]. In addition, MRI-detected tenosynovitis has been shown to predict both radiographic and MRI damage progression in RA patients in clinical remission [36]. The impact of patient-reported flares on long-term radiographic and structural MRI outcomes was beyond the scope of the present study.

The interval between FV1 and FV2 was based on previous observations that the majority of patient-reported flares were transient and lasted less than 1 week [2, 42]. EULAR guidelines for patients with active RA recommend tight monitoring every 1–3 months [43]. Since we considered patient-reported flare as a proxy for disease activity, the third follow-up visit (FV3) was scheduled after approximately 3 months, when we expected that the hand flare had resolved. The last follow-up visit (FV4), which took place 7–10 days after FV3, served as a comparator to FV3 to investigate whether outcomes changed within approximately 1 week, when the disease was stable and patients did not report a flare. We did not find differences in outcomes obtained 7–10 days apart, except for PGA which can be affected by factors not related to RA itself, such as psychological distress or comorbidity [44].

Strengths of our study were the prospective data collection, very low attrition rates, and pre-specified serial imaging assessments. All clinical, laboratory, and imaging procedures along with collection of PROs were performed on the same day at each visit, except in four patients, who underwent MRI the day after the other examinations due to logistic reasons. Furthermore, we utilized and validated reliable definitions and scoring systems for imaging modalities. The same rheumatologist scored all US scans with high reliability [30]. MRI evaluations were conducted by two readers who displayed very good agreement, and the images were read blinded to chronological order.

Some limitations merit comment. The flare definition was based on an anchor question and a condition of at least one tender and swollen joint, as perceived by the patient. This definition does not cover the full spectrum of patient-reported flares [7]. We may speculate that some patients did not contact the clinic in case of a hand flare being aware of the consequences of four time-consuming clinical visits, a factor which would contribute to underestimation of the flare occurrence. Moreover, the threshold when to contact the clinic might have been set high by the patients themselves, resulting in reporting only flares of higher intensity. However, average flare severity at flare visit was 4.8 (2.7) which is lower than recently observed in a large cohort of RA patients, who scored a median (IQR) flare severity of 7 [5, 8] [45].

The relative small sample size may be a limitation. However, the sample of 29 patients was not pre-specified. Among the patients who met the pre-defined inclusion criteria (*n* = 80), all patients who contacted the hospital because of a self-reported hand flare (*n* = 29) were included in the present study. The lack of a comparator group may be another limitation, as we do not have data on temporal fluctuations in imaging findings in patients who did not report a flare. However, extensive serial imaging examinations in patients in stable remission or low disease activity who do not report a flare are not intuitive from the perspective of clinical routine.

The results of the sensitivity analyses presented in Additional file 4: Table S4 with flare duration as an explanatory variable needs to be interpreted with caution due to imbalanced frequencies of observations across the four categories and especially a very small number of observations in the category of 8–14 days (*n* = 2).

## Conclusions

In conclusion, the clinical signature of patient-reported flares aligned with imaging-defined inflammation on MRI and US of the hands. Patient-reported flares were associated with imaging biomarkers of pathophysiological manifestations of RA, i.e., inflammation in the synovium and tenosynovium, and in case of long-lasting flares, also in the bone marrow. We observed a differential sequence of tissue inflammation in relation to flare dynamics. Synovitis on MRI and US as well as tenosynovitis on US appeared early at flare onset but were short-lived. By contrast, BME evolved with delay and persisted after the flare had resolved, while MRI tenosynovitis increased rapidly and remained elevated for months. Our findings indicate that patient-reported flares reflect the inflammatory burden of a relapse, which calls for a re-appraisal of patient-reported inflammation to drive the management of patients with RA.

## Supplementary information

**Additional file 1: Table S1.** Imaging biomarkers at baseline and third follow-up visit (FV3) in patients who did not report a new flare, after the hand flare had resolved (*n* = 25).

**Additional file 2: Table S2.** Inter-reader agreement for MRI read-out.

**Additional file 3: Table S3.** Intra-reader agreement for MRI read-out based on re-scoring of 5 out of 29 patients.

**Additional file 4: Table S4.** Associations between imaging biomarkers and flare duration.

## Data Availability

The data that support the findings of this study are not publicly available and restrictions apply to the availability of these data according to the Danish Data Protection Regulation. Data are however available from the authors upon reasonable request and with permission of the University of Southern Denmark, legal services of the Research & Innovation Organization and approval from the Danish Data Protection Agency.

## Abbreviations

3D: Three dimensional
ACR: American College of Rheumatology
Anti-CCP: Anti-cyclic citrullinated peptide
bDMARD: Biological disease-modifying anti-rheumatic drug
BME: Bone marrow edema
CD: Color Doppler
CI: Confidence interval
CRP: C-reactive protein
csDMARDs: Conventional synthetic disease-modifying anti-rheumatic drug
DAS28CRP: Disease Activity Score for 28 joints based on CRP
EGA: Evaluator global assessment
EULAR: European League Against Rheumatism
FLARE-RA: Flare Rheumatoid Arthritis Questionnaire
FOV: Filed of view
FV: Follow-up visit
GD: Gadoteric acid contrast
GE: Gradient echo
GLOESS: Global OMERACT-EULAR synovitis score
GS: Gray scale
HAQ: Health assessment questionnaire
i.a: Intra-articular
ICC: Intra-class correlation coefficient
LDA: Low disease activity
MCP: Metacarpophalangeal
MRI: Magnetic resonance imaging
OMERACT: Outcome Measures in Rheumatology
PD: Power Doppler
PGA: Patient global assessment of disease activity
PIP: Proximal interphalangeal
PRF: Pulse repetition frequency
PRO: Patient-reported outcome
RA: Rheumatoid arthritis
RA-FQ: OMERACT rheumatoid arthritis flare questionnaire
RAMRIS: Rheumatoid arthritis magnetic resonance imaging score
RF: Rheumatoid factor
SD: Standard deviation
SDC: Smallest detectable change
SJC28: Swollen joint count in 28 joints
ST: Slice thickness
STIR: Short-tau inversion recovery
T1-w: T1-weighted
TE: Echo time
TJC28: Tender joint count in 28 joints
TR: Repetition time
US: Ultrasonography
VAS: Visual analog scale
