# Supplementary material for: Serial magnetic resonance imaging and ultrasound examinations demonstrate differential inflammatory lesion patterns in soft tissue and bone upon patient-reported flares in rheumatoid arthritis
Source: Arthritis Res Ther. 2020 Feb 3;22:19. doi: 10.1186/s13075-020-2105-6 (PMC6998154; doi:10.1186/s13075-020-2105-6)
Supplement: Supplementary file 1 — Additional file 1: Table S1. Imaging biomarkers at baseline and third follow-up visit (FV3) in patients who did not report a new flare, after the hand flare had resolved (n = 25). [file 13075_2020_2105_MOESM1_ESM.docx]

**Supplementary table 1.** Imaging biomarkers at baseline and third follow-up visit (FV3) in patients who did not report a new flare, after the hand flare had resolved (n=25)

______________________________________________________

| Characteristic | Baseline | FV3 | p-value |
| --- | --- | --- | --- |
| ____________________________________________________ | | | |
| MRI synovitis | 10 (4.1) | 10.6 (3.6) | 0.35 |
| MRI tenosynovitis | 6.1 (4.8) | 7.5 (5.3) | 0.02 |
| MRI BME | 2.9 (5.1) | 2.9 (3.5) | 0.01* |
| US synovitis (GLOESS) | 8.0 (5.4) | 9.1 (4.2) | 0.28 |
| US tenosynovitis | 3.2 (4.6) | 4.2 (5.2) | 0.18 |

______________________________________________________

BME, bone marrow edema; FV, follow-up visit; GLOESS, Global OMERACT (Outcome Measures in Rheumatology)-EULAR (European League Against Rheumatism) Synovitis Score; MRI, magnetic resonance imaging; US, ultrasonography

*Wilcoxon signed-rank test was applied to accommodate non-normally distributed BME data
