# Supplementary material for: Serial magnetic resonance imaging and ultrasound examinations demonstrate differential inflammatory lesion patterns in soft tissue and bone upon patient-reported flares in rheumatoid arthritis
Source: Arthritis Res Ther. 2020 Feb 3;22:19. doi: 10.1186/s13075-020-2105-6 (PMC6998154; doi:10.1186/s13075-020-2105-6)
Supplement: Supplementary file 2 — Additional file 2: Table S2. Inter-reader agreement for MRI read-out. [file 13075_2020_2105_MOESM2_ESM.docx]

**Supplementary table 2.** Inter-reader agreement for MRI read-out

____________________________________________________________________________

|  | Status scores | | | Change scores |
| --- | --- | --- | --- | --- |
|  | Baseline  ICC (95%CI) | Follow-up visit 1  ICC (95%CI) | Aggregate score across all 5 time points*  ICC (95%CI) | Baseline to follow-up visit 1  ICC (95%CI) [SDC] |
| ____________________________________________________________________________ | | | | |
| Synovitis | 0.85  (0.70;0.93) | 0.82  (0.66;0.91) | 0.82  (0.64;0.91) | 0.86  (0.73;0.93) [1.71] |
| Tenosynovitis | 0.86  (0.72;0.93) | 0.88  (0.77;0.94) | 0.87  (0.74;0.94) | 0.94  (088.;0.97) [1.38] |
| BME | 0.96  (0.92;0.98) | 0.91  (0.83;0.96) | 0.94  (0.87;0.97) | 0.88  (0.75;0.94) [1.68] |

BME, bone marrow edema, 95%CI: 95%Confidence interval, ICC: Intra-class correlation coefficient, MRI, magnetic resonance imaging; SDC: Smallest detectable change;

*5 time points: baseline, follow-up visit 1- 4
