# Supplementary material for: Serial magnetic resonance imaging and ultrasound examinations demonstrate differential inflammatory lesion patterns in soft tissue and bone upon patient-reported flares in rheumatoid arthritis
Source: Arthritis Res Ther. 2020 Feb 3;22:19. doi: 10.1186/s13075-020-2105-6 (PMC6998154; doi:10.1186/s13075-020-2105-6)
Supplement: Supplementary file 3 — Additional file 3: Table S3. Intra-reader agreement for MRI read-out based on re-scoring of 5 out of 29 patients. [file 13075_2020_2105_MOESM3_ESM.docx]

**Supplementary table 3.** Intra-reader agreement for MRI read-out based on re-scoring of 5 out of 29 patients

___________________________________________________________________________

|  | Status scores | | | Change scores |
| --- | --- | --- | --- | --- |
|  | Baseline  ICC (95% CI) | Follow-up visit 1  ICC (95% CI) | Aggregate score across all 5 time points* | Baseline to Follow-up Visit 1  ICC (95% CI)  [SDC] |
| _________________________________________________________________________ | | | | |
| *Rater 1* |  |  |  |  |
| Synovitis | 0.95  (0.04;1.0) | 0.99  (0.96;1.0) | 0.95  (0.18;1.0) | 0.84  (0.03;0.95)  [0.43] |
| TS | 0.88  (0.22;0.99) | 0.97  (0.81;1.0) | 0.88  (0.30;0.99) | 0.86  (0.46;0.99)  [1.78] |
| BME | 0.99  (0.92;1.0) | 1 | 0.99  (0.95;1.0) | 0.98  (0.88;1.0)  [1.48] |
| *Rater 2* |  |  |  |  |
| Synovitis | 0.87  (0.29;0.98) | 0.88  (0.36;0.98) | 0.89  (0.29;0.99) | 0.87  (0.25;0.99)  [1.20] |
| TS | 0.86  (0.28;0.98) | 0.95  (0.67;0.99) | 0.87  (0.30;0.98) | 0.95  (0.67;0.99)  [0.87] |
| BME | 0.99  (0.95;1.0) | 0.93  (0.56;0.99) | 0.95  (0.66;0.99) | 0.96  (0.75;1.0)  [1.38] |

BME, bone marrow edema, 95%CI: 95% Confidence interval, ICC: Intra-class correlation coefficient, MRI, magnetic resonance imaging; SDC: Smallest detectable change, TS, tenosynovitis;

*5 time points: baseline, follow-up visit1- 4
