# Supplementary material for: Serial magnetic resonance imaging and ultrasound examinations demonstrate differential inflammatory lesion patterns in soft tissue and bone upon patient-reported flares in rheumatoid arthritis
Source: Arthritis Res Ther. 2020 Feb 3;22:19. doi: 10.1186/s13075-020-2105-6 (PMC6998154; doi:10.1186/s13075-020-2105-6)
Supplement: Supplementary file 4 — Additional file 4: Table S4. Associations between imaging biomarkers and flare duration. [file 13075_2020_2105_MOESM4_ESM.docx]

**Supplementary table 4.** Associations between imaging biomarkers and flare duration

________________________________________________________________________________

|  | | Univariate model | | Adjusted model* | |
| --- | --- | --- | --- | --- | --- |
| Outcome | Flare duration  (days) | Coefficient (95%CI) | p | Coefficient (95%CI) | p |
| ______________________________________________________________________________ | | | | | |
| MRI synovitis | 4-7 | 0.61 (-1.36,2.58) | 0.54 | 0.82 (-1.18,2.83) | 0.42 |
|  | 8-14 | -0.53 (-2.01,0.95) | 0.48 | -0.41 (-1.91,1.09) | 0.59 |
|  | >14 | 2.70 (0.88,4.53) | 0.004 | 2.78 (0.94,4.61) | 0.003 |
| MRI tenosynovitis | 4-7 | 0.90 (-1.74,3.54) | 0.50 | 0.98 (-1.67,3.64) | 0.47 |
|  | 8-14 | -1.16 (-3.03,0.71) | 0.23 | -1.08 (-2.95,0.80) | 0.26 |
|  | >14 | 1.83 (-0.54,4.20) | 0.13 | 1.97 (-0.39,4.33) | 0.10 |
| MRI BME | 4-7 | 0.21 (-0.04,.47) | 0.10 | 0.20 (-0.05,0.45) | 0.12 |
|  | 8-14 | 0.12 (-0.05,0.29) | 0.18 | 0.11 (-0.06,0.28) | 0.21 |
|  | >14 | 0.28 (0.06,0.50) | 0.012 | 0.27 (0.05,0.49) | 0.016 |
| US synovitis  (GLOESS) | 4-7 | -1.07 (-4.76,2.62) | 0.57 | -1.51 (-5.09,2.07) | 0.41 |
|  | 8-14 | 1.96 (-0.98,4.9) | 0.19 | 1.96 (-0.94,4.87) | 0.19 |
|  | >14 | 3.63 (0.12,7.14) | 0.04 | 3.49 (0.14,6.85) | 0.04 |
| US tenosynovitis | 4-7 | 1.68 (-1.35,4.71) | 0.28 | 1.88 (-1.18,4.94) | 0.23 |
|  | 8-14 | 0.60 (-1.56,2.76) | 0.58 | 0.68 (-1.49,2.85) | 0.54 |
|  | >14 | -0.26 (-2.99,2.46) | 0.85 | -0.22 (-2.95,2.52) | 0.88 |

________________________________________________________________________________

Mixed effects linear regression with flare duration category 1-3 days as base. *Adjusted for: age, disease duration at baseline and sex.

BME, bone marrow edema; GLOESS, Global OMERACT (Outcome Measures in Rheumatology)-EULAR (European League Against Rheumatism) Synovitis Score; MRI, magnetic resonance imaging; US, ultrasonography.
